# Supplementary figures and images for: PP2A-Cdc55 phosphatase regulates actomyosin ring contraction and septum formation during cytokinesis
Source: Cell Mol Life Sci. 2022 Mar 1;79(3):165. doi: 10.1007/s00018-022-04209-1 (PMC8888506; doi:10.1007/s00018-022-04209-1)

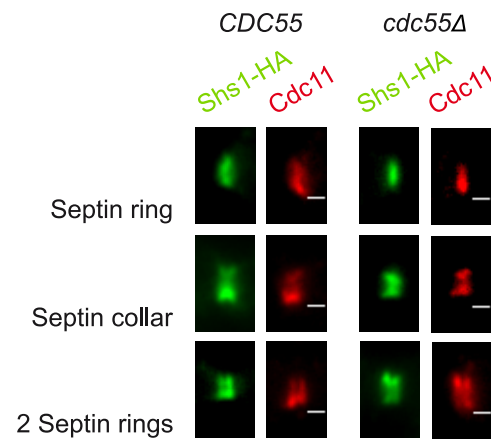

Supplement: Supplementary file 1 — Supplementary file1 Septin structures are not altered in the absence of Cdc55. Cycling cells from strains Y1588 and Y1589 were fixed and immunofluorescence in situ performed for septins Cdc11 and Shs1-HA visualization. α-Cdc11 and α-HA (12CA5) antibodies were used. Representative images of Cdc11 and Shs1 septin’ structures from CDC55 and cdc55Δ cells are shown. Scale bar, 1 μm. (PDF 47 KB) [file 18_2022_4209_MOESM1_ESM.pdf]

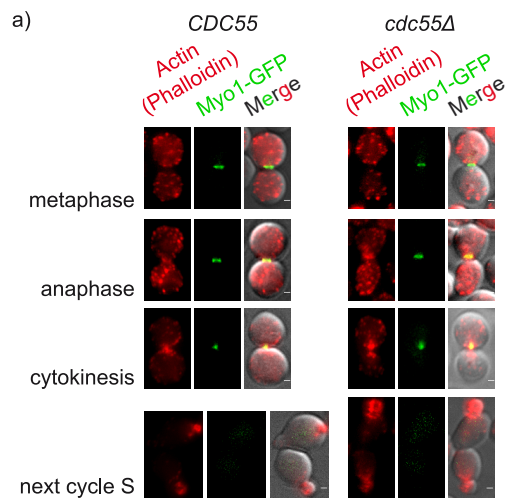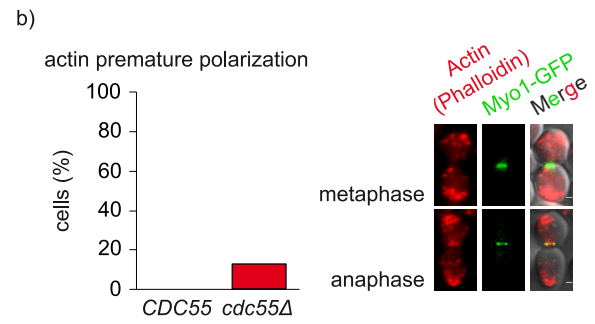

Supplement: Supplementary file 2 — Supplementary file2 Actin is polarized to the new bud site before cytokinesis completion in cdc55Δ cells. Strains Y1434 and Y1435 were arrested in metaphase by Cdc20 depletion and released into anaphase by Cdc20 re-induction. Formaldehyde-fixed cells were stained with 50 U/mL of rhodamine-phalloidin. (a) Representative images of the actin staining and Myo1-GFP signals from CDC55 and cdc55Δ at different cell cycle stages (metaphase, anaphase, cytokinesis and the next S phase) are shown. (b) Quantifications and representative images of the cells with re-polarized actin at the new bud site are shown. Scale bar, 1 μm. (PDF 313 KB) [file 18_2022_4209_MOESM2_ESM.pdf]

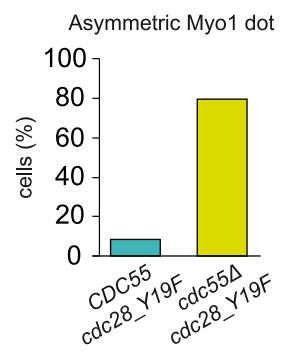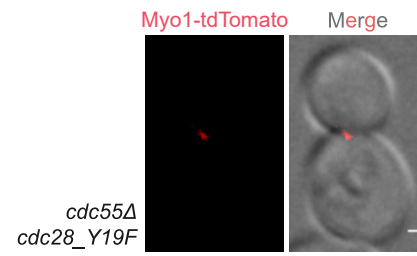

Supplement: Supplementary file 3 — Supplementary file3 Myo1 asymmetry contraction in G1-synchronized cells in absence of Cdc55. Strains Y1631 (N=15) and Y1761 (N=12) were arrested in G1 by α-factor addition and released into cell cycle progression by pheromone removal. Cells were fixed with formaldehyde before taking images. Quantifications of the cells with asymmetric Myo1 contraction are represented (left panel). Representative images of Myo1 asymmetric contraction are shown (right panel). Scale bar, 1 μm. (PDF 50 KB) [file 18_2022_4209_MOESM3_ESM.pdf]

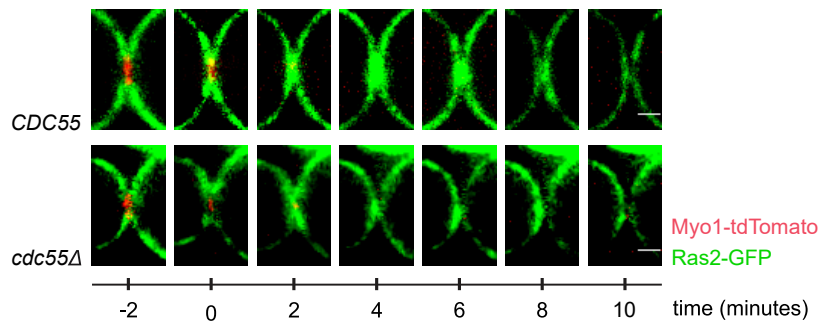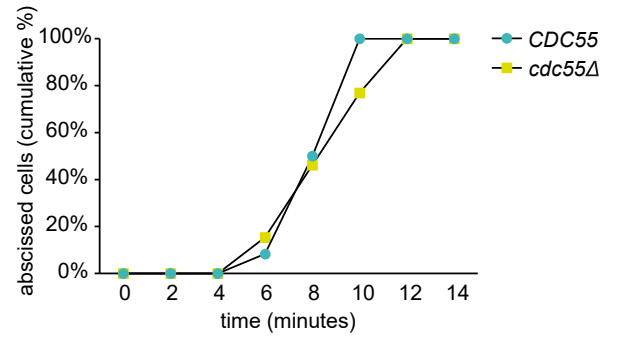

Supplement: Supplementary file 4 — Supplementary file4 Proper cytoplasm separation during membrane abscission in the absence of Cdc55. Strains Y1717 and Y1708 were arrested in metaphase by Cdc20 depletion and released into anaphase by Cdc20 re-induction. Time-lapse images were captured every 2 minutes. Membrane abscission was followed by visualization of the GFP3-Ras2 signal, and Myo1-tdTomato was used as a control for cytokinesis progression. Representative images from CDC55 (N=12) and cdc55Δ (N=13) cells are shown (left panel). Images are taken from the best z-stack to visualize the division site. Scale bar, 1 μm. Quantification of the cumulative percentage of cells abscised is represented (right panel). (PDF 60 KB) [file 18_2022_4209_MOESM4_ESM.pdf]

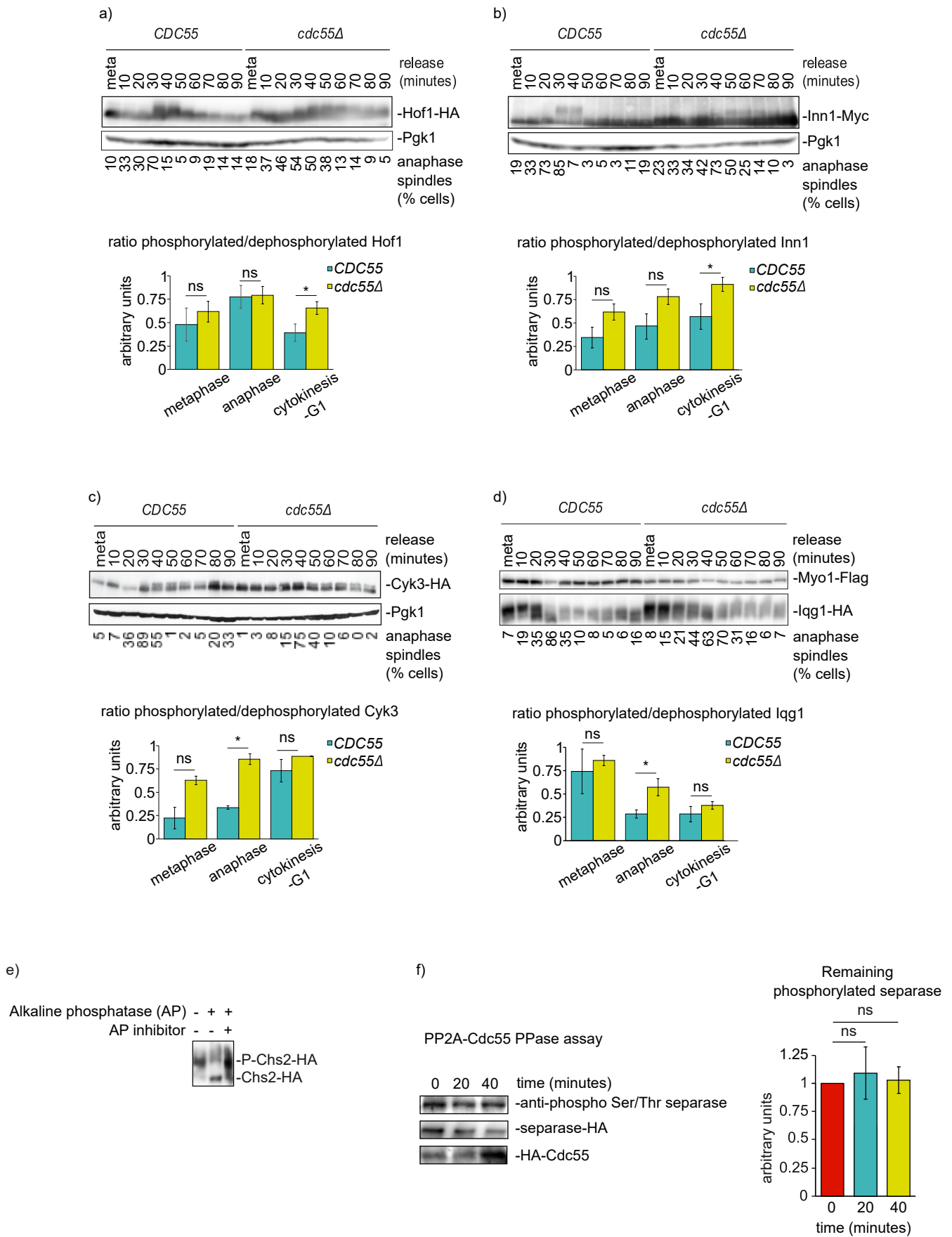

Supplement: Supplementary file 5 — Supplementary file5 PP2A-Cdc55 participates in the dephosphorylation of IPCs proteins during cytokinesis. (a-d) Phosphorylation changes of Hof1, Inn1, Cyk3 and Iqg1 in absence of Cdc55. Strains Y1314, Y1394, Y1639, Y1640, Y1437, Y1438, Y1491 and Y1497 were arrested in metaphase and released into anaphase by Cdc20 depletion and re-addition. Protein phosphorylations were analyzed by western blot. Pgk1 and Myo1-Flag levels were used as a loading control. Mitosis progression was followed by analyzing the anaphase spindle elongation by in situ immunofluorescence. At least 100 cells were scored at each time point. Quantifications of the western blots were performed using Fiji Software and means and SEMs are represented. Student’s unpaired t-test analyses were carried out using the Prism5 program. (e) Alkaline phosphatase assay for Chs2. Native protein extracts were prepared from Y1318 cells arrested in metaphase and incubated with alkaline phosphatase and alkaline phosphatase’s inhibitor as indicated. PhosStop was used as the alkaline phosphatase’s inhibitor. Chs2 phosphorylation was analyzed by western blot in Phos-tag gels. (f) Separase is not dephosphorylated by PP2A-Cdc55. Metaphase arrested cells of the strain Y695 were used to purify the PP2A-Cdc55 complex by TAP purification. Esp1-HA was purified from metaphase-arrested cells of strain Y1748. Purified Esp1-HA was incubated with the PP2A-Cdc55 complex at the indicated times and the separase phosphorylation levels were detected by western blot using the anti-phospho Ser/Thr antibody. Representative images of one phosphatase assay are shown. Protein levels were quantified using Fiji software. Quantifications of the remaining separase phosphorylation signal normalized to the amount of Cdc55 and Esp1 are shown. Means and SEMs of three phosphatase assays are represented. Student’s unpaired t-test analysis was carried out using the Prism5 program. (PDF 693 KB) [file 18_2022_4209_MOESM5_ESM.pdf]

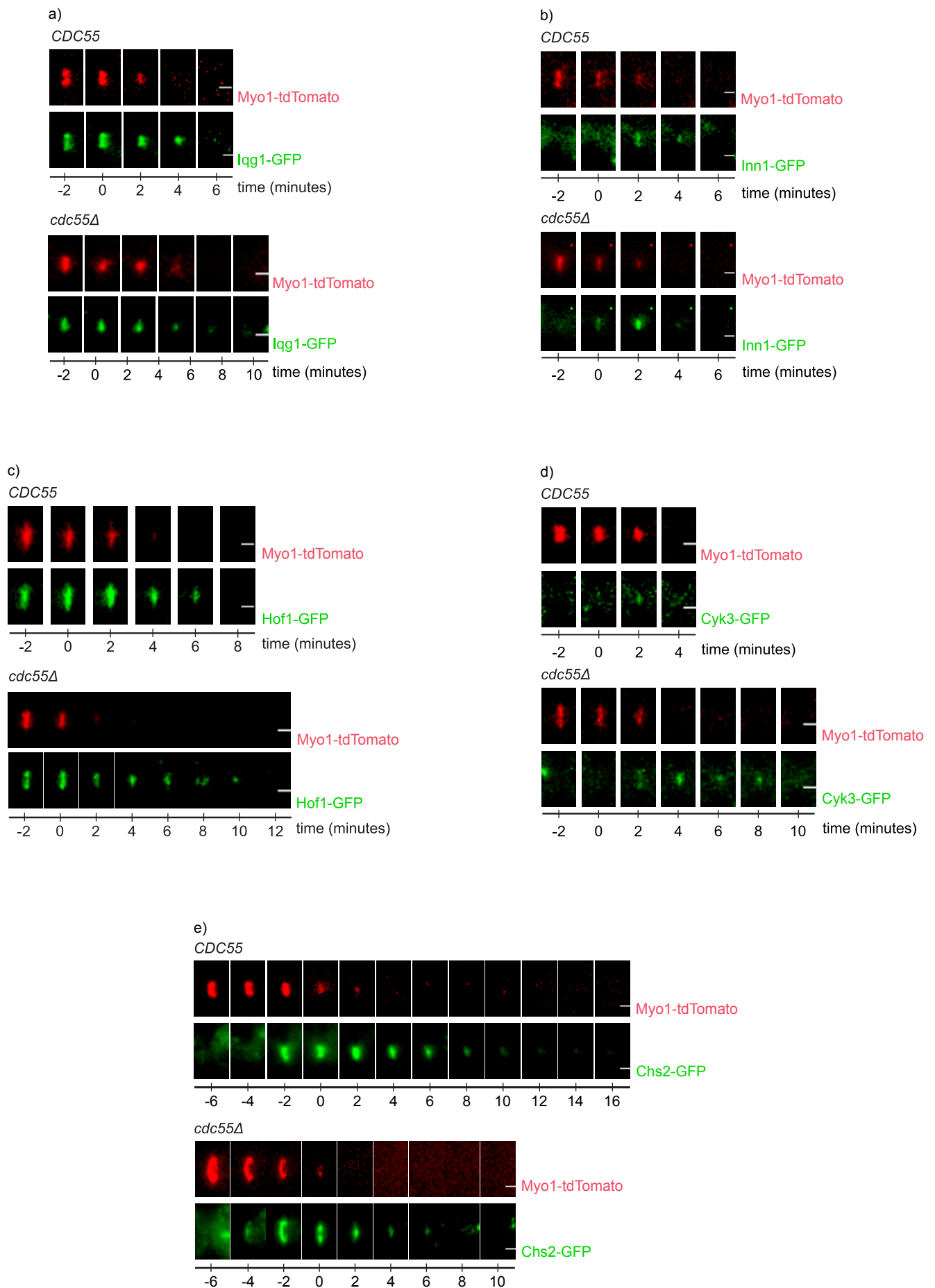

Supplement: Supplementary file 6 — Supplementary file6 PP2A-Cdc55 is required for proper IPCs localization and contraction at the division site. Strains Y1572, Y1606, Y1454, Y1608, Y1306, Y1578, Y1574, Y1604, Y1576 and Y1575 were synchronized into anaphase by Cdc20 depletion and re-addition, and time-lapse images were captured every 2 minutes. Myo1-tdTomato was used as a control for cytokinesis progression. (a-e) Representative images of the indicated GFP-tagged IPCs proteins and Myo1 contractions from CDC55 and cdc55Δ cells are shown. Iqg1 and Inn1 contraction at the bud neck are normal in the absence of Cdc55. By contrast, Hof1 and Cyk3 contractions are longer in the absence of Cdc55. Scale bar, 1 μm. (PDF 167 KB) [file 18_2022_4209_MOESM6_ESM.pdf]
