# Supplementary material for: PP2A-Cdc55 phosphatase regulates actomyosin ring contraction and septum formation during cytokinesis
Source: Cell Mol Life Sci. 2022 Mar 1;79(3):165. doi: 10.1007/s00018-022-04209-1 (PMC8888506; doi:10.1007/s00018-022-04209-1)
Supplement: Supplementary file 7 — Supplementary file7 (PDF 37 KB) [file 18_2022_4209_MOESM7_ESM.pdf]

Table S1. List of strains used in this study

Strain	Genotype	Origin
W303	*MATa ade2-1 trp1-1 can1-100 leu2-3,112 his3-11,15 ura3 GAL psi+*	Matt Sullivan
Y695	*MATa CDC14-Pk9 TAP-HA-CDC55 GAL1-CDC20*	This study
Y824	*MATα MET-HA3-CDC20*	This laboratory
Y844	*MATα cdc55Δ*	This laboratory
Y1306	*MATα MET-HA3-CDC20 MYO1-tdTOMATO SPC42-GFP HOF1-GFP*	This study
Y1314	*MATa MET-HA3-CDC20 HOF1-HA6*	This study
Y1315	*MATα MET-HA3-CDC20 CHS2-HA6 cdc55Δ*	This study
Y1318	*MATa MET-HA3-CDC20 CHS2-HA6*	This study
Y1394	*MATα MET-HA3-CDC20 HOF1-HA6 cdc55Δ*	This study
Y1434	*MATa MET-HA3-CDC20 MYO1-GFP cdc55Δ*	This study
Y1435	*MATa MET-HA3-CDC20 MYO1-GFP*	This study
Y1437	*MATa MET-HA3-CDC20 CYK3-HA6*	This study
Y1438	*MATa MET-HA3-CDC20 CYK3-HA6 cdc55Δ*	This study
Y1454	*MATa MET-HA3-CDC20 MYO1-tdTOMATO SPC42-GFP INN1-GFP*	This study
Y1491	*MATa MET-HA3-CDC20 MYO1-FLAG IQG1-HA6 HOF1-MYC9*	This study
Y1497	*MATα MET-HA3-CDC20 MYO1-FLAG IQG1-HA6 cdc55Δ*	This study
Y1512	*MATa MET-HA3-CDC20 MYO1-tdTOMATO SPC42-GFP cdc55Δ*	This study
Y1516	*MATa MET-*HA_3*-CDC20 CHS2-GFP SPC42-GFP*	This study
Y1567	*MATa MET-HA3-CDC20 CHS2-HA6 PK3-CDC55*	This study
Y1572	*MATa MET-HA3-CDC20 MYO1-tdTOMATO SPC42-GFP yE-GFP-IQG1*	This study
Y1574	*MATa MET-HA3-CDC20 MYO1-tdTOMATO SPC42-GFP CYK3-GFP*	This study
Y1575	*MATa MET-HA3-CDC20 MYO1-tdTOMATO CHS2-GFP cdc55Δ*	This study
Y1576	*MATα MET-HA3-CDC20 MYO1-tdTOMATO SPC42-GFP CHS2-GFP*	This study
Y1578	*MATa MET-HA3-CDC20 MYO1-tdTOMATO HOF1-GFP cdc55Δ*	This study
Y1588	*MATa MET-HA3-CDC20 SHS1-HA6*	This study
Y1589	*MATa MET-HA3-CDC20 SHS1-HA6 cdc55Δ*	This study
Y1596	*MATα MET-HA3-CDC20 MYO1-tdTOMATO chs3Δ cdc55Δ*	This study
Y1604	*MATa MET-HA3-CDC20 MYO1-tdTOMATO SPC42-GFP CYK3-GFP cdc55Δ*	This study
Y1605	*MATα MET-HA3-CDC20 MYO1-tdTOMATO chs3Δ*	This study
Y1606	*MATa MET-HA3-CDC20 MYO1-tdTOMATO SPC42-GFP yE-GFP-IQG1 cdc55Δ*	This study
Y1608	*MATa MET-HA3-CDC20 MYO1-tdTOMATO SPC42-GFP INN1-GFP cdc55Δ*	This study
Y1631	*MATa MYO1-tdTOMATO CDC15-eGFP cdc55Δ cdc28-Y19F*	This study
Y1639	*MATa MET-HA3-CDC20 CYK3-HA6 INN1-Myc9*	This study
Y1640	*MATa MET-HA3-CDC20 CYK3-HA6 INN1-Myc9 cdc55Δ*	This study
Y1652	*MATa MET-HA3-CDC20 MYO1-tdTOMATO cdc55Δ URA3::HA3-CDC55*	This laboratory
Y1653	*MATa MET-HA3-CDC20 MYO1-tdTOMATO cdc55Δ URA3::HA3-CDC55-T174E_S301D*	This laboratory
Y1708	*MATa MET-HA3-CDC20 MYO1-tdTOMATO 3GFP-RAS2 cdc55Δ*	This study
Y1717	*MATa MET-HA3-CDC20 MYO1-tdTOMATO 3GFP-RAS2*	This study

Y1728	*MATa ADH1-AtTIR1-MYC9 hof1-aid*	Sánchez-Díaz A laboratory
Y1730	*MATa GAL1-UBR1 ADH1-AtTIR1-MYC9 td-cyk3-aid*	Sánchez-Díaz A laboratory
Y1747	*MATa GAL1-UBR1 ADH1-AtTIR1-MYC9 td-cyk3-aid cdc55Δ*	This study
Y1748		
Y1749	*MATa ADH1-AtTIR1-MYC9 hof1-aid cdc55Δ*	This study
Y1761	*MATa MYO1-tdTOMATO CDC15-eGFP cdc28-Y19F*	This study
Y1788	*MATα MET-HA3-CDC20 MYO1-tdTOMATO SPC42-GFP pGPD1-OsTIR1-MYC9 cdc55-aid*	This study
Y1893	*MATa MET-HA3-CDC20 MYO1-tdTOMATO SPC42-GFP GAL-CHS2-S133E-YFP*	This study
Y1894	*MATa MET-HA3-CDC20 MYO1-tdTOMATO SPC42-GFP GAL-CHS2-S133E-YFP cdc55Δ*	This study
Y1895	*MATa MET-HA3-CDC20 MYO1-tdTOMATO SPC42-GFP GAL-CHS2-YFP*	This study
Y1896	*MATa MET-HA3-CDC20 MYO1-tdTOMATO SPC42-GFP GAL-CHS2-YFP cdc55Δ*	This study
Y1898	*MATa MET-HA3-CDC20 MYO1-tdTOMATO SPC42-GFP GAL-CHS2-S133A-YFP cdc55Δ*	This study
Y1900	*MATa MET-HA3-CDC20 MYO1-tdTOMATO GAL-CHS2-S133A-YFP*	This study
Y1901	*MATa MET-HA3-CDC20 MYO1-tdTOMATO GAL-CHS2-YFP*	This study
Y1909	*MATa MET-HA3-CDC20 HOF1-mCherry SPC42-GFP GAL-CHS2-YFP*	This study
Y1910	*MATa MET-HA3-CDC20 HOF1-mCherry SPC42-GFP GAL-CHS2-S133A-YFP*	This study
Y1911	*MATa MET-HA3-CDC20 HOF1-mCherry SPC42-GFP GAL-CHS2-S133E-YFP*	This study
Y1912	*MATa MET-HA3-CDC20 HOF1-mCherry SPC42-GFP GAL-CHS2-YFP cdc55Δ*	This study
Y1913	*MATa MET-HA3-CDC20 HOF1-mCherry SPC42-GFP GAL-CHS2-S133A-YFP cdc55Δ*	This study
